# Red Shift in the Absorption Spectrum of Phototropin LOV1 upon the Formation of a Semiquinone Radical: Reconstructing the Orbital Architecture

**DOI:** 10.1021/acs.jpcb.4c00397

**Published:** 2024-04-30

**Authors:** Patrick Kurle-Tucholski, Christian Wiebeler, Lisa Köhler, Ruonan Qin, Ziyue Zhao, Mantas Šimėnas, Andreas Pöppl, Jörg Matysik

**Affiliations:** † Institut für Analytische Chemie, Universität Leipzig, Linnéstraße 3, D-04103 Leipzig, Germany; ‡ Institut für Physik, Universität Augsburg, Universitätsstraße 1, D-86159 Augsburg, Germany; § Faculty of Physics, Vilnius University, Sauletekio 3, LT-10257 Vilnius, Lithuania; ∥ Felix Bloch Institute for Solid State Physics, Universität Leipzig, Linnéstraße 5, D-04103, Leipzig, Germany

## Abstract

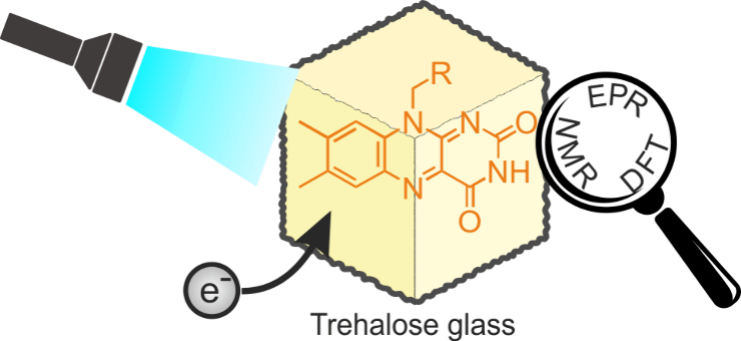

Flavin mononucleotide (FMN) is a ubiquitous blue-light pigment due to its ability to drive one- and two-electron transfer reactions. In both light-oxygen-voltage (LOV) domains of phototropin from the green algae *Chlamydomonas reinhardtii*, FMN is noncovalently bound. In the LOV1 cysteine-to-serine mutant (C57S), light-induced electron transfer from a nearby tryptophan occurs, and a transient spin-correlated radical pair (SCRP) is formed. Within this photocycle, nuclear hyperpolarization is created by the solid-state photochemically induced dynamic nuclear polarization (photo-CIDNP) effect. In a side reaction, a stable protonated semiquinone radical (FMNH^·^) forms undergoing a significant bathochromic shift of the first electronic transition from 445 to 591 nm. The incorporation of phototropin LOV1-C57S into an amorphous trehalose matrix, stabilizing the radical, allows for application of various magnetic resonance experiments at ambient temperatures, which are combined with quantum-chemical calculations. As a result, the bathochromic shift of the first absorption band is explained by lifting the degeneracy of the molecular orbital energy levels for electrons with alpha and beta spins in FMNH^·^ due to the additional electron.

## Introduction

**Figure 1:**
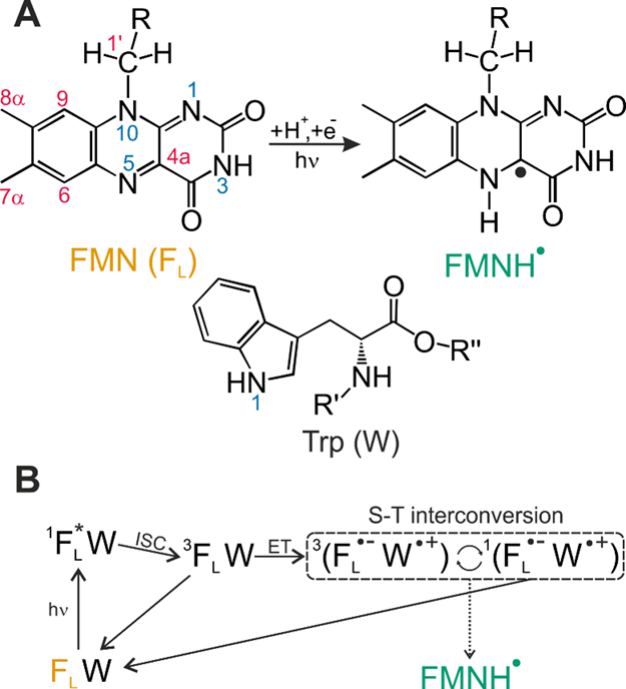
(A) Chemical structure of FMN (F_L_, yellow) with its relevant atom positions, with R representing the ribityl side chain. Reduction and subsequent protonation lead to a semiquinone radical (FMNH^·^, turquois). Shown below is the structure of tryptophan with its one-letter code W. (B) Simplified photocycle of phototropin LOV1 C57S from *C. reinhardtii*. Light excitation forms the singlet excited state of F_L_ which undergoes intersystem crossing (ISC) to a long-lived molecular triplet. Subsequent electron transfer from tryptophan W98 forms a spin-correlated radical pair (SCRP) coherently oscillating coherently between the electronic triplet and singlet states inducing photo-CIDNP nuclear hyperpolarization which can be detected with NMR. Furthermore, byproduct FMNH^·^ is formed by a side reaction quitting the photo-CIDNP photocycle.

Flavins exhibit remarkable functional versatility as cofactors, participating in a myriad of molecular processes such as DNA repair or phototropism.^1^ Flavoproteins are also discussed to allow for animal navigation in the earth’s magnetic field.^2^ The common motif of flavins is the isoalloxazine ring structure, which, in the case of flavin mononucleotide (FMN), is substituted at the nitrogen position 10 with a ribityl side chain. Its functional diversity is due to different catalytically active oxidation states which can mediate both one- and two-electron transfer processes often coupled to proton transfer.^3^ Upon one-electron reduction followed by protonation, a semiquinone radical (FMNH^·^) is formed, as depicted in Figure 1 A. Extensive investigations employing a variety of spectroscopic techniques and quantum chemical calculations have shed light on the properties of FMN and FMNH^·^, both within flavoproteins and as isolated systems.^4−9^

FMN takes a critical role as a chromophore for function in light-oxygen-voltage (LOV) sensing domains, where it acts as a blue-light pigment, governing light-activated phototropic processes in plants and algae.^10^ Within phototropin from the green algae *Chlamydomonas reinhardtii*, two LOV domains, triggered by blue light absorption, regulate an N-terminal serine/threonine kinase.^11,12^ Here, FMN is noncovalently bound inside both LOV domains. Upon light excitation and subsequent intersystem crossing (ISC), FMN generates a molecular triplet state (^3^FMN), which then leads to the formation of a single bond to a nearby cysteine residue.^13^ By replacement of the cysteine with serine or alanine through mutagenesis, the lifetime of the molecular triplet state is extended, resulting in the formation of a spin-correlated radical pair (SCRP) upon electron-transfer from a tryptophan (W98).^13^ The SCRP evolves coherently in the singlet–triplet manifold leading to nuclear hyperpolarization via a solid-state photochemically induced dynamic nuclear polarization (photo-CIDNP) effect, which is detected by nuclear magnetic resonance (NMR) as a dramatic increase in signal intensity.^14−17^ The SCRP can decay to the electronic ground state by recombination into the singlet state, completing the photocycle. An observed concurrent side reaction is the formation of the radical FMNH^·^. A simplified reaction scheme is shown in Figure 1B. The formation of the radical FMNH^·^ can be optically followed due to a bathochromic shift (i.e., red shift) of the first electronic transition from 445 to 591 nm (see below). Compared to an aromatic molecule of a similar size as anthracene, having the first absorption maximum at 375 nm, the question of the orbital architecture in FMN and FMNH^·^ allows for absorption at such long wavelengths. One might expect, for example, the involvement of n-π* transitions. The present work is dedicated to the elucidation of the orbital architecture of FMN and its protonated radical.

During light irradiation, the surrounding protein undergoes photodegradation due to the formation of singlet oxygen.^18−21^ Recently, stabilization and protection of flavin-containing proteins by embedding in an amorphous matrix of the disaccharide trehalose has been reported.^22^ The sugar matrix still allowed for observation of photo-CIDNP enhancement in solids at room temperature while prolonging the effective measurement time significantly from minutes to hours. It has been demonstrated that incorporation into trehalose matrices dramatically slows down processes such as electron transfer reactions^23,24^ or light-induced chromophore changes,^25^ while maintaining the protein structure.^26^

The ability of trehalose glass for protein stabilization in combination with the possibility of undisturbed light penetration, as well as the protection against singlet oxygen, makes it a prime candidate for investigations of light-induced processes in flavoproteins. Here, we show that trehalose-embedding can be used for photo-CIDNP MAS NMR experiments at ambient temperatures to provide direct insight into photochemistry. Furthermore, the observation of a stable semiquinone radical was confirmed by optical absorption spectroscopy.^27^ Advantageously, the sample can be stored at ambient temperatures for months as an amorphous powder,^25^ making handling of the sample easier in comparison to proteins in buffer solution. We also show that semiquinone can be characterized using electron paramagnetic resonance (EPR) and ^1^H electron nuclear double resonance (ENDOR) spectroscopy.

Our findings suggest that trehalose matrices are suitable for the stabilization of proteins, which enables the investigation of different spectroscopic methods at ambient temperatures. Based on this condition, the experimental characterization of two oxidation states of flavin, here FMN and FMNH^·^, becomes possible. The combination of spectroscopic data with theoretical studies offers insight into the orbital architecture and absorption properties.

## Experimental Methods and Computational Details

### Protein Expression

Phototropin-LOV1-C57S (*Cr*LOV1) from the unicellular algae *C. reinhardtii* was expressed from a plasmid carrying an N-terminal 10x His-tag.^28^ For UV/vis and EPR spectroscopy, *Cr*LOV1 was expressed in *Escherichia coli* strain BL21 (DE3) using LB-medium. The protein for^15^N photo-CIDNP experiments was expressed in M9 minimal medium with ^15^N isotope-enriched ^15^NH_4_Cl.^17^ Both types are purified by Ni^2+^-affinity chromatography (kta purifier, GE Healthcare). Residual imidazole was removed by dialysis against phosphate buffer (300 mM NaCl and 50 mM KH_2_PO_4_/K_2_HPO_4_, pH 8.0). About 20 mg of protein was concentrated using an ultracentrifugation filter with a molecular cutoff at 10 kDa (Merck Millipore, MWCO 10000). The appropriate volume of trehalose solution (1.2 M stock solution) was added to the concentrated protein sample achieving a molar ratio of 50:1 (trehalose to protein). The mixture of trehalose and *Cr*LOV1 was dried on a Petri dish under nitrogen gas for several hours until a solid amorphous glass was formed.

### Photo-CIDNP MAS NMR

Photo-CIDNP magic-angle spinning (MAS) experiments were carried out on a 4.7 T NMR spectrometer (Bruker, Karlsruhe, Germany) operating at 200.13 MHz proton frequency (^15^N frequency at 20.28 MHz) equipped with a 3.2 mm double resonance NMR probe. Both samples containing *Cr*LOV1 in buffer solution as well as embedded inside trehalose were packed in different 3.2 mm sapphire rotors. The trehalose sample was then measured at room temperature while the sample inside the buffer solution was frozen at 264 K with 500 Hz spinning to ensure homogeneous sample distribution. The trehalose sample was spun at an 8 kHz spinning frequency, whereas for the frozen solution, a spinning frequency of 12 kHz was applied. For 1D photo-CIDNP MAS NMR experiments, an echo sequence with an optimized 90° hard pulse for ^15^N of 3.75 μs and SW_f_-TPPM heteronuclear decoupling^29^ at 100 kHz was applied during acquisition. The spectral width was 14705 Hz with a relaxation delay of 3 s. In each case, 40 scans were recorded. For photo-CIDNP MAS NMR, the sample was irradiated with a 488 nm continuous wave (CW) laser (GENESIS MX488–1000 STM OPS-Laser-Diode System, Coherent Europe B.V., The Netherlands) operating at a 1 W output power. For photo-CIDNP SUPER MAS NMR experiments,^30^ the rotors were spun at 4 kHz resulting in an effective rf field of 48.48 kHz for CSA recoupling. The γ-integral was set to one, and 64 scans were recorded in each of the 24 t_1_-increments. The spectral width was set to 14,705 Hz taking the scaling factor of 0.155 into account.^31^ Heteronuclear SW_f_-TPPM decoupling was used during t_1_ and t_2_ acquisition, and frequency discrimination was achieved using the STATES-TPPI method.^32^ Determination of the principal values involved automated fitting with ssNake,^33^ while the CSA tensors were simulated using SIMPSON.^30,34−36^

### UV/Vis Absorption Spectroscopy

Absorption experiments were performed on a Shimadzu 1900i spectrometer at ambient temperature. The background of the Petri dish was removed before drying the sample. After the trehalose-protein mixture was dried on the Petri dish, a dark state absorption spectrum was recorded. Then, the sample was irradiated with a 488 nm continuous wave (CW) laser, which was also used for photo-CIDNP experiments. Here, the sample was placed inside a box lined with aluminum foil, in order to enhance the effect of stray light reaching the sample, and the laser beam was adjusted onto a specific spot of the Petri dish with an optical fiber. After an irradiation of about 1 min, a local change of color was observed, and again, a UV/vis spectrum was recorded.

### EPR

CW X-band (∼9.5 GHz) EPR spectra were measured at a Bruker EMXmicro spectrometer fitted with a Bruker ER4119HS cylindrical cavity. The CW Q-band (∼34 GHz) EPR spectrum was recorded using a Bruker EMX 10–40 spectrometer equipped with a cylindrical cavity.

### ENDOR

^1^H-ENDOR spectra at X-band were recorded with the Davies ENDOR pulse sequence (37) at a Bruker ELEXYS E580 spectrometer using an EN 4118X-MD4 Bruker resonator. Microwave (mw) pulse lengths *t*_π/2_ = 0.1 μs and *t*_π_ = 0.2 μs and a radiofrequency (rf) pulse length *t*_πRF_ = 10 μs, together with the mw pulse delay τ = 1 μs, were employed. For ^1^H Davies ENDOR experiments at the Q-band (~34 GHz), a Bruker ELEXSYS E580/IF-Q EPR spectrometer equipped with an EN5107D2 Bruker mw resonator was used. The mw pulse lengths were *t*_π/2_ = 16 ns and *t*_π_ = 32 ns and an rf pulse length was *t*_πRF_ = 12 μs. The mw pulse delay of τ = 0.3 μs was used. We also performed ^1^H-ENDOR experiments at Q-band using the Mims () pulse sequence.^38^ The Mims ^1^H-ENDOR was obtained by summing the spectra recorded by varying the interpulse delay τ from 120 to 400 ns. All ENDOR experiments were performed at 90 K, and the temperature was controlled using an Oxford Instruments CF935 cryostat. The ENDOR spectra were recorded at the center of the radical EPR spectrum at magnetic field values of 348.4 mT (X-band) and 1211.5 mT (Q-band) corresponding to the *g*-value of *g* = 2.0038. The EPR and ENDOR data were simulated by MATLAB R2019b using the EasySpin toolbox (version 6.0.0-dev36).^39^

### Theoretical Calculations

The initial structures of FMN and Trp were taken from our recent publication,^22^ in which the ribityl side chain of FMN was replaced by a methyl group. For FMNH^·^, we added a proton at the corresponding position and performed calculations for the charge-neutral doublet state. In the case of FMN and Trp, calculations were realized for their neutral singlet forms. All quantum chemical calculations employed Orca version 5.0.3.^40^ For the optimizations, TPSSh^41,42^/cc-pVTZ^43^ together with the conductor-like polarizable continuum model (CPCM) with water as solvent was used as the level of theory. The obtained structures were verified to be minima on the potential energy surface by performing frequency calculations at the same level of theory and finding only positive vibrational frequencies. The newly optimized structures of FMN and FMNH^·^ can be found in the Supporting Information.

These three structures were the starting points for the subsequent determination of excited states as well as of hyperfine coupling and absolute shielding constants, which were realized employing similar levels of theories as for optimizations, i.e., TPSSh as functional and CPCM with water as solvent. The first 10 excited states of FMN and FMNH^·^ were determined with time-dependent density functional theory (TD-DFT) and using aug-cc-pVTZ^44^ as the basis set. For calculating the hyperfine coupling constants of FMNH^·^, we employed the property-optimized pcH-3 basis set,^45^ which was retrieved from the basis set exchange database.^46^ For the absolute shielding constants of FMN and Trp, we utilized the pcSseg-3 basis.^47^ For the latter two kinds of calculations, we required a very tight SCF convergence. In the case of the shielding calculations, we also employed a denser integration grid (defgrid3) and the RIJCOSX approximation together with the default auxiliary basis sets. Subsequently, the absolute shielding constants from our calculations were correlated with the experimental chemical shifts. The parameters derived from linear regression were then used to convert the calculated shielding constants into chemical shifts for both isotropic values and principal components of the tensors.

## Results

**Figure 2:**
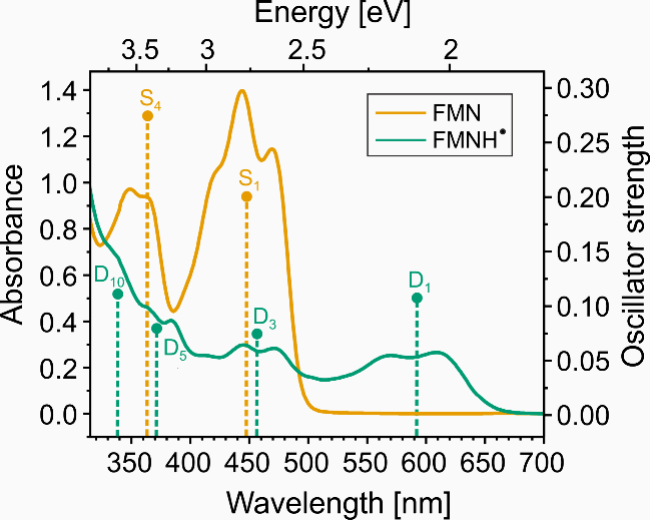
UV/vis absorption spectrum of phototropin LOV1 C57S in a glassy trehalose matrix on a Petri dish. The signal background of the Petri dish was removed by a blank measurement. Measurements were conducted at the same spot with the flavin in its oxidized form (yellow) and after 488 nm irradiation with a cw-laser (green) forming FMNH^·^. At shorter wavelengths, the scattering background is clearly visible. Excitation energies and oscillator strengths for the brighter transitions from quantum chemical calculations are shown as dotted sticks, and the inset indicates the two kinds of states.

UV/vis absorption spectroscopy was employed to investigate the FMN chromophore in *Cr*LOV1 embedded inside an amorphous glassy trehalose matrix at room temperature. The obtained absorption spectrum, depicted in Figure 2 (yellow), exhibits two characteristic absorption bands at 348 and 445 nm, corresponding to π → π* transitions of FMN.^48^ Quantum chemical calculations (see Table S1) show that relatively bright transitions named S_1_ and S_4_ are found within the wavelength ranges of the two absorption bands. According to the calculations, the transitions named S_2_, S_3_ and S_5_ exhibit weak oscillator strengths. Therefore, these calculations agree well with the experimentally obtained spectrum. Besides, the liquid-state absorption spectra of *C. reinhardtii* LOV1 measured between 350 and 700 nm (data not shown) are nearly identical to the spectrum, as shown in Figure 2. Hence, the stabilization of the LOV domain by the trehalose matrix does not compromise the chromophore absorption properties. More specifically, the glassy matrix facilitates light penetration and absorption in solid amorphous powders, thereby promoting light-induced processes as applied in a variety of spectroscopical methods.^49^ Furthermore, compared to proteins in aqueous buffer solution, embedding inside trehalose has the ability to accommodate high protein loading about 10 times higher compared to a frozen solution,^25^ allowing for an increased signal-to-noise ratio upon spectroscopic observation. This advantage is particularly valuable for spectroscopic methods such as nuclear magnetic resonance (NMR)^25,50,51^ and in particular, solid-state photo-CIDNP NMR experiments,^22^ which greatly benefit from significantly enhanced sensitivity.

**Figure 3:**
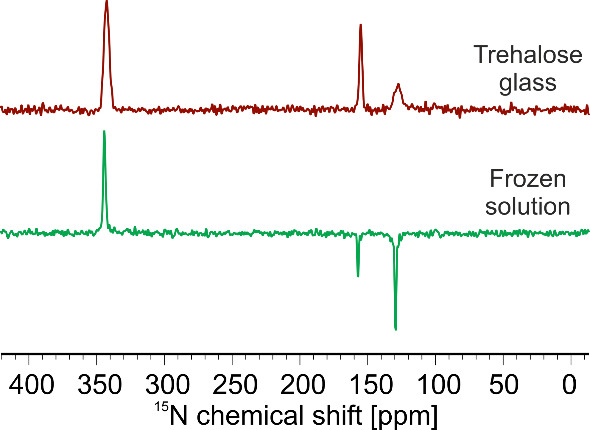
^15^N solid-state photo-CIDNP spectra of u-[^15^N] CrLOV1 C57S in a trehalose matrix at room temperature (red, top) and in a frozen solution at 235 K (green, bottom) at 4.7 T (200 MHz ^1^H frequency). Three light-induced signals are observed at 342 ppm (assigned to FMN-N5), 156 ppm (FMN-N10), and 129 ppm (W98-N1).

Figure 3 shows solid-state photo-CIDNP MAS NMR spectra of [u-^15^N]-labeled *Cr*LOV1 C57S in both trehalose glass (top) and frozen solution (bottom). In both spectra, the same three light-induced signals are observed and can be assigned to N5 (342 ppm) and N10 (156 ppm) of FMN as well as to N1 (129 ppm) of tryptophan W98. While the chemical shifts of the signals remain consistent, some notable differences are observed. First, a sign change of FMN-N10 and W98-N1 from negative, i.e., emissive (frozen solution), to positive, i.e., enhanced absorptive (trehalose glass), occurs. This seems to be a feature of trehalose-embedded *Cr*LOV1, as has been shown earlier, and has been speculated to be related to the shortening of the SCRP lifetime affecting the spin dynamics.^22^ For example, in a trehalose-embedded photosynthetic reaction center, radical pair recombination is reduced by a factor of 2 compared to a water-glycerol matrix.^24^ Second, the peak width of W98-N1 is significantly broadened in trehalose glass compared to frozen solution. This might be attributed to the stabilization effect of trehalose leading to a more rigid protein matrix and fixing the donor W98 moiety in various conformations.^52^ In contrast, the noncovalently bound FMN is not as much affected at the N10 position. The signal assigned to N5, however, exhibits a broadening from 39 Hz (fwhm) in frozen solution to 93 Hz in trehalose. In Table 1, the isotropic chemical shift, the relative sign, and the line width of each photo-CIDNP enhanced signal are summarized. Photo-CIDNP-enhanced signals in solids offer a significant amount of information, which can be obtained by specific experiments.^30,53−55^ While chemical shifts are related to the local electron spin density in the electronic ground state after the photocycle, three-spin-mixing (TSM)-caused intensities correspond to the local electron spin density in the radical pair state, while differential relaxation (DR)-caused intensities are related to the local electron spin density in the molecular triplet state.^53^

**Table 1:** Isotropic Chemical Shift, the Relative Sign (*A* stands for Absorptive, and *E* stands for Emissive), and the Line Width for FMN-N5, N10, and W98-N1, Which Are Photo-CIDNP Enhanced, Are Summarized

position		frozen solution		trehalose	
	δ_iso_ (ppm)	sign	FWHM (Hz)	sign	FWHM (Hz)
FMN-N5	342	*A*	39	*A*	93
FMN-N10	156	*E*	26	*A*	48
W98-N1	129	*E*	29	*A*	130

**Figure 4:**
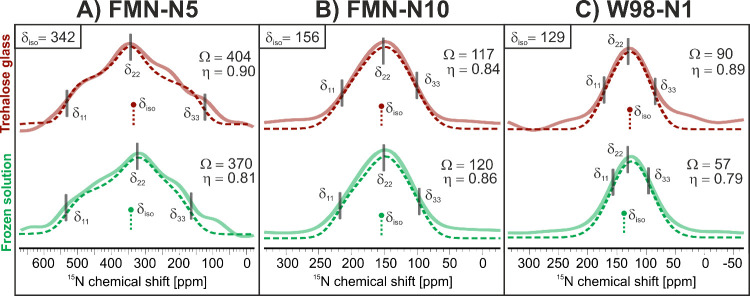
Cross sections (solid lines) from ^15^N photo-CIDNP SUPER MAS NMR experiments in trehalose glass (red, top) and in the frozen solution (green bottom) for (A) FMN-N5 (δ_iso_ = 342 ppm), (B) FMN-N10 (δ_iso_ = 156 ppm), and (**C**) W98-N1 (δ_iso_ = 129 ppm). Fit curves obtained from simulations are shown as dotted lines. The principal values (δ_11_, δ_22_, δ_33_) as well as the span (Ω [ppm]) and the asymmetry factor (η) are extracted from fitting and displayed in Table S3.

Figure 4 depicts the chemical shift anisotropy (CSA) tensors extracted from the 2D photo-CIDNP SUPER MAS NMR experiment conducted on *Cr*LOV1 in a frozen solution (green) and within a trehalose matrix (red). The principal values of the tensors as well as the values for the span and its asymmetry parameter are presented. Detailed fitting parameters are shown in Table S3. Generally, the shape of the CSA tensors remains largely unchanged in both matrices for all three signals. Specifically, for FMN-N5 (Figure 4, spectrum A), a broad CSA pattern with large anisotropy is observed in both cases, with the span and asymmetry parameters exhibiting similar values. The tensor shape suggests that FMN-N5 is sp^2^-hybridized^56,57^ in its electronic ground state and connects to a large conjugated π-system within the isoalloxazine ring. In contrast, FMN-N10 (Figure 4B) shows a narrow, relatively symmetric tensor, as indicative of sp^3^ hybridization. The CSA tensor of FMN-N10 remains unaffected by the protein matrix, suggesting minimal influence from the protein environment on the cofactor at this specific position. The CSA tensor of W98-N1 (Figure 4C) also displays such a symmetrical shape. This implies that the nitrogen of the quasi-aromatic pyrrole ring of the indole exhibits sp^3^ hybridization that might be attributed to a reduced cyclic conjugation.^58^ However, compared to the frozen solution, the principal values are altered in trehalose glass. Especially, the slightly larger span, considering the uncertainty of the fitting parameters, might hint at a decreased dynamics of W98, similar to those observed in the broad peak in Figure 3. Besides, it must be noted that the experimentally determined spans are smaller in comparison to the quantum-chemical calculations (see Table S4). This indicates that the molecular model used for the calculations presents a more rigid structure and environment, which is not surprising, as the calculations of FMN and Trp were performed for one optimized structure only.

Upon solid-state photo-CIDNP MAS NMR experiments, a noticeable change in color from yellow to green/blue was observed in the powdered sample. Therefore, we irradiated the Petri dish containing the glassy amorphous trehalose-protein mixture with a 488 nm CW laser reproducing this color change (see Figure S1). Absorption spectroscopy reveals a red shift of the main absorption band as depicted in Figure 2 (turquoise), which is characteristic of FMNH^·^.^59,60^ The lowest energy absorption band is now located at 591 nm and is attributed to a π → π* transition.^48^ Quantum-chemical calculations show that this absorption is caused by transition D_1_, whose energy is in good agreement with the position of the experimental absorption band. Interestingly, a residual absorption band at 445 nm was still observed, which can be attributed either to the second transition of FMNH^·^ or an incomplete conversion from FMN to FMNH^·^. Going to even lower wavelengths, some shoulders in the absorption spectra might be associated with transitions D_5_ and D_10_, as these transitions are relatively intense and found close to the positions expected from the experimental spectra.

**Figure 5:**
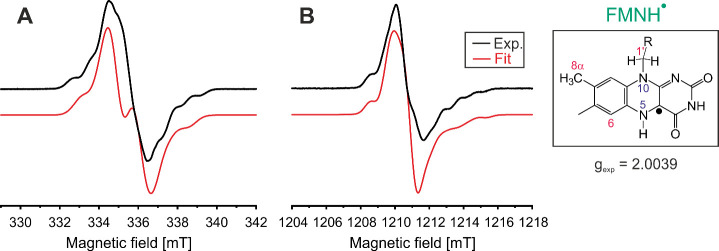
X- (A) and Q-band (B) CW EPR spectra of *Cr*LOV1 at room temperature after the formation of FMNH^·^. The experimentally determined *g*-factor is 2.0039. The simulated fit curve is shown in red, taking into account *g* = [2.0044 2.0038 2.0019]^4^ and the HFCs of N5, N10, and H5 shown in Table 2.

For further characterization of FMNH^·^ in trehalose glass, X- (Figure 5A) and Q-band (Figure 5B) CW EPR experiments were conducted at room temperature. The *g*-factor was determined to be 2.0039. The line width and line shape are mainly dominated by ^1^H and ^14^N hyperfine couplings (HFC) of the isoalloxazine moiety, while a second radical, although expected, could not be detected. However, the spectral resolution of the CW EPR spectra is not sufficient to determine the principal values of the HFC tensors of the various ^1^H and ^14^N nuclei of the isoalloxazine moiety from the CW EPR powder patterns. Therefore, both experimental CW EPR spectra were simulated with the HFC parameters obtained from the quantum-chemical computation with small adjustments (Figure 5), as summarized in Table 2. Our simulation reveals that the hyperfine structure observed is due to large anisotropic HFCs from nitrogen N5 as well as proton H5. The signs of the hyperfine couplings cannot be experimentally determined and are obtained from theoretical calculations. Here, the large A_*z*_ component of N5 and H5 is characteristic of FMN, as described in previous studies.^4^ In addition, HFC to N10 can be identified, contributing to the hyperfine structure with a large A_*z*_ component. Overall, anisotropic HFCs are the dominating factors in the isoalloxazine moiety. Although satisfactory agreement between experimental and simulated CW EPR spectra was achieved, a reliable determination of all principal values of the ^1^H HFC tensors is not feasible. This is particularly important for H5 and H8α as their HFCs indicate the formation of an FMNH^·^ radical.^61^

**Table 2:** Hyperfine Couplings of FMNH^·^ Experimentally Determined, by Fitting Using the Parameters from Quantum Chemical Calculations as Starting Points (see Table S5) from CW EPR and ^1^H ENDOR Spectra

atom position	hyperfine couplings (MHz)				method applied
	*A*_*x*_	*A*_*y*_	*A*_*z*_	*A*_iso_	
N5	–1.79	–1.92	47.16	14.48	CW EPR
N10	0.09	–0.21	26.32	8.73	CW EPR
H1′	8.00	8.00	8.00	8.00	ENDOR
H5	–1.87	–26.76	–39.00	–22.54	ENDOR/CW EPR
H6	–1.59	–5.80	–5.80	–4.39	ENDOR
H8α	6.80	6.80	8.00	7.20	ENDOR

**Figure 6:**
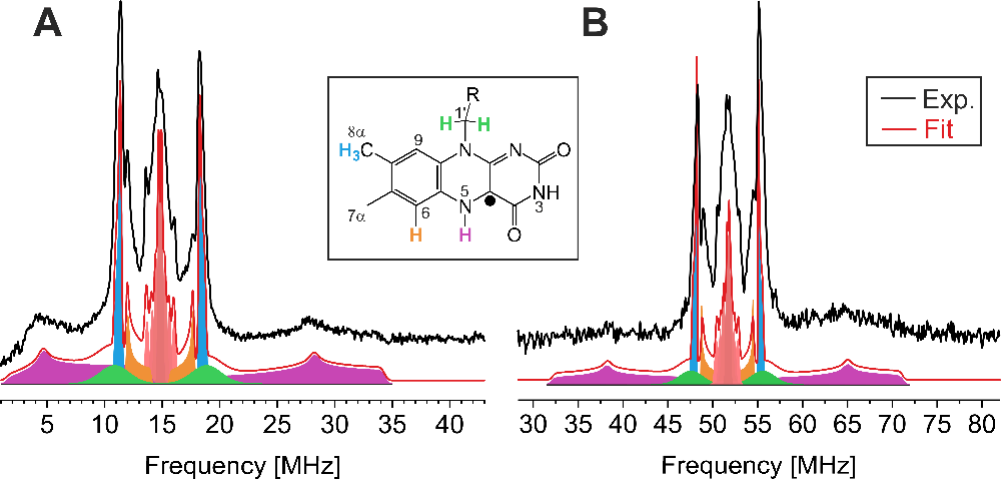
X-band (A) and Q-band (B) experimental ^1^H Davies ENDOR spectra of FMNH^·^ at 90 K (black line). The fitting curves result from HFC from the assigned protons of FMN (H1′ (green), H5 (purple), H-6 (orange), and H8α (blue) as well as from small HFC contributions (red) related to H7α and H9 and the surrounding protein matrix. HFC data are presented in Table 2.

To extract these proton HFCs from FMNH^·^, ^1^H Davies ENDOR experiments at 90 K were conducted at X- (Figure 6A) and Q-band (Figure 6B) frequencies. The powder-like spectra including spectra simulations are presented in Figure 6. For spectral analysis, the results of the quantum chemical calculations were used as starting parameters to obtain the proton HFCs and their individual contributions to the line shape, as depicted with different colors in the fit. The determined values of the HFCs are summarized in Table 2.

The red-highlighted region in the center of the spectra corresponds to the various small HFCs (<2.0 MHz) from the weakly coupled protons H7α and H9 as well as from amino acids of nearby residues and surrounding water molecules.^4^ Next, a pair of anisotropic signals (orange) can be seen and is assigned to H6. A slightly larger HFC is observed (blue), which can be assigned to the three protons H8α of the methyl group.^62^ Underlying the methyl group signal, isotropic broad signals (green) are observed originating from one of the two protons H1′ belonging to the side chain. Finally, a broad anisotropic peak is seen (purple). Due to spectral folding in the X-band ENDOR spectrum, the signal to the left of the center is observed at 1–14 MHz, while its partner transition shows a signal at 16–35 MHz. The spectrum in Figure 6B, however, is not affected by spectral folding; thus, both partner transitions of the large anisotropic peak can be assigned to H5, as it has been reported for FMNH^·^ in flavoproteins.^63,64^ Q-band Mims ENDOR spectra fully support the analysis of the Davies ENDOR spectra (Figure S3).

## Discussion

### ^15^N Photo-CIDNP MAS NMR of u^15^N-LOV1 in Trehalose Matrix

Trehalose glass provides a unique matrix for spectroscopic investigations of proteins at room temperature. Its ability to stabilize the protein backbone and simultaneously keep dynamic processes intact, albeit at a reduced rate, in conjunction with its limited light scattering characteristic, can be utilized to study photodynamic processes such as electron transfer^22^ or photoswitching.^25,51^*Cr*LOV1 incorporated into trehalose glass is a prime candidate for NMR studies, exploiting the solid-state photo-CIDNP effect and creating hyperpolarized signals. Our earlier study^22^ demonstrated its feasibility to study hyperpolarized^13^C signals in a flavoprotein at natural abundance. In the present study, we show solid-state photo-CIDNP MAS NMR spectra obtained from uniformly ^15^N enriched *Cr*LOV1 in both a frozen buffer solution and a solid trehalose matrix (see Figure 3). The three light-induced signals have been assigned to both participating electron transfer partners forming the SCRP, namely, the acceptor FMN and the donor W98. The constancy of the chemical shift in both matrices demonstrates that the protein stabilized in trehalose preserves its structural integrity and maintains its ability to undergo photochemistry. However, there are noticeable differences between the spectra obtained in frozen solution and in trehalose, in particular the sign change of a signal, whose origin remains unclear, and the broadening of the peaks from the FMN-N5 and W98-N1 nitrogens. The broadening is caused by the fixation of different conformations. At the FMN, N5 is affected and not N10. It might be that N10 is fixed by its side chain in any case. Upon trehalose treatment, the signal of W98-N1 disintegrates into several components, and the span of the CSA tensor increases (Figure 4). Here, upon band fitting, the component with the highest intensity is shown, as the other two components exhibit similar parameters (see Table S5). It is not unexpected that the aromatic amino acid W98, which is localized on the protein surface, undergoes conformational changes. The data suggest that several conformations, which are frozen in trehalose, are photo-CIDNP-active. Hence, the local dynamics of the tryptophan side chain is reduced in trehalose compared to the frozen solution. It can be concluded that the stabilization primarily occurs at the interface between the protein and amorphous trehalose. This supports the anchorage model,^65^ which states that the protein is anchored within a high-rigidity matrix. Residual water predominantly surrounds the protein, forming a hydration layer that is closely connected to both the protein as well as the amorphous part of the trehalose matrix. The cofactor is most likely affected by the reduced amount of water inside the protein pocket. The relevance of the amorphous part of trehalose for stabilization has been shown by MAS NMR studies on the aging process in trehalose-embedded phytochromes.^25^ In that case, the conversion of amorphous to crystalline trehalose was related to the effective stabilization of the embedded proteins. Interestingly, the chromophore is not as much influenced by the stabilization process as the tryptophan residue. This has similarly been observed in photosynthetic reaction centers embedded in a trehalose matrix.^66^ Here, the motional dynamics of quinone Q_A_ in its anionic radical state after electron transfer from the special pair remained the same and was not influenced by the changes in the local environment. The authors concluded that the observed change in radical-pair recombination rate originates from increased rigidity of the surrounding protein due to its surface interaction with the dry trehalose glass.

From an experimental perspective, the utilization of trehalose-embedded protein samples offers several distinct advantages when compared to frozen solutions. First, it affords a substantial increase in the loaded protein quantity by approximately a factor of 10.^25^ This enhancement in protein loading inherently increases the signal-to-noise ratio. In combination with isotope enrichment as well as solid-state photo-CIDNP MAS NMR, it provides a suitable tool to investigate flavoproteins. Second, the experiments can be conducted at ambient temperatures, which more closely resemble natural conditions. Additionally, handling of the powdered sample becomes more manageable in terms of rotor packing, achieving stable spinning conditions as well as executing experiments under ambient temperature conditions. At last, in contrast to NMR experiments on ^13^C, detection of ^15^N does not yield the signals of the trehalose matrix and solely provides information on the protein signals. More specifically, in our case, only light-induced signals appear, further simplifying the data interpretation. Nevertheless, the sign change of the photo-CIDNP enhanced signals remains unclear. An alternation of the triplet lifetime of FMN or the radical pair lifetime might be the driving factor. Therefore, time-resolved photo-CIDNP as well as time-dependent absorption studies could offer insight into the underlying spin dynamics.

### Formation of the FMNH^·^ Radical

Photo-CIDNP MAS NMR experiments focused on the electronic ground-state state of the FMN after the photocycle. There is, however, a side reaction leading to the product FMNH^·^ which has been studied in the past at liquid nitrogen temperatures.^4,61^ Interestingly, this radical is stable for several months in trehalose and can be characterized at ambient temperatures using various EPR techniques. The stability of FMNH^·^ might be due to a lack of reaction partners, such as oxygen or water, in the immediate vicinity. It has to be noted that neither EPR nor ^1^H ENDOR identified a second radical species, which is consistent with earlier findings.^4,62,63,67^ Additionally, the absorption spectrum does not show an absorption at 500 nm, which would be characteristic of the tryptophanyl radical.^68^ This absence is quite surprising, considering that the solid-state photo-CIDNP effect relies on the formation of a radical pair that clearly involves W98. The subsequent formation of FMNH^·^ appears to coincide with the disappearance of the second radical whose fate remains unclear.

### Comparison of Spectroscopic Data between FMN and the FMNH Radical

**Figure 7:**
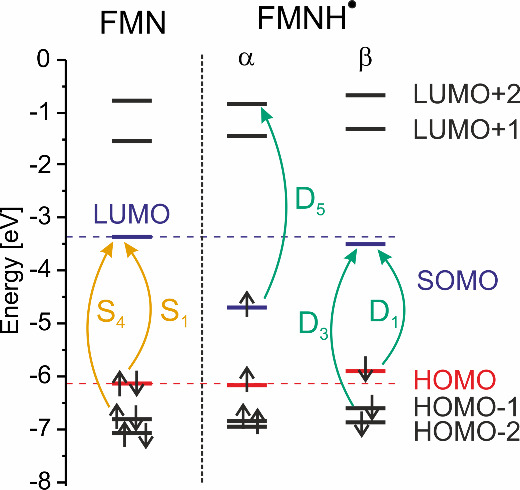
Scheme of frontier molecular orbitals for FMN and FMNH^·^. In the case of the latter, orbitals for the two spins are shown separately. For FMN, the lowest electronic transition corresponds to the excitation of an electron from HOMO to LUMO (S_1_), whereas the second intense transition is an excitation from HOMO-1 to LUMO (S_4_). All three molecular orbitals involved exhibit π character; see also Figure S2 with the depiction of the relevant MOs. In case of FMNH^·^, energy levels for the two spin states of the same orbital are shown separately. The first two transitions with oscillator strengths larger than 0.05 (D_1_, D_3_) are similar in nature to the ones in FMN, as the involved orbitals for the β spin resemble the orbitals for the closed shell FMN (S_1_, S_4_). In contrast to this, the third transition (D_5_) is dominated by the excitation of an electron with α spin from SOMO to LUMO+2.

The bathochromic shift of the absorption band upon radical formation from FMN to FMNH^·^ toward higher wavelengths is remarkably large. Figure 7 shows the energy-level diagram for both species including their individual transitions, denoted as in the absorption spectra in Figure 2. The absorption is largely determined by the conjugated π-system (see Figure S2) which, in particular, includes the benzene ring as well as the N5–C4a–C10a–N1 motif. Remarkably, there are also strong contributions from N10, despite its formal sp^3^ hybridization, which would be expected to lower the aromaticity of the system. The two most intense electronic transitions of FMN are from the highest occupied molecular orbital (HOMO) to the lowest unoccupied molecular orbital (LUMO), denoted as S_1_ (“S” refers to the singlet spin state), and from HOMO-1 to the LUMO, denoted as S_4_. These transitions give rise to the two main absorption bands at 448 (2.76 eV) and 364 nm (3.40 eV). Both bands are also observed experimentally (Figure 2). A similar good agreement between excited state calculations and the measured absorption spectrum is also obtained for FMNH^·^. The first transition, D_1_ at 592 nm (2.10 eV; “D” refers to the doublet spin state), lies in the first absorption band, whereas the second transition with significant oscillator strengths is D_3_ at 456 nm (2.72 eV), and it falls within the second absorption band. Furthermore, the next intense transitions, D_5_ at 371 nm (3.34 eV) and D_10_ at 339 nm (3.66 eV), coincide with shoulders in the experimental absorption spectrum. It has to be noted that the lowest energy absorption bands exhibit vibrational fine structures with additional maxima at 420 and 469 nm for FMN and 575 and 610 nm for FMNH^·^. These fine structures appear to be related to some stretching modes of the fused rings, which are found around 1200 cm^–1^.^69^ To further investigate this, we also performed calculations taking vibronic effects into account, and the results can be found in the Supporting Information. Furthermore, computational studies that took vibronic effects into account have been reported.^69,70^ However, the calculation of vertical excitations reported in the article is sufficient for the discussion of the electronic transitions that are contributing toward the absorption spectrum. Furthermore, we have found that the molecular orbitals involved in the bright electronic transitions are rather independent of the functional as long as PCM is used, whereas, for example, the excitation energies quite strongly depend on the functional; see the Supporting Information.

Therefore, we are now discussing the change in absorption upon radical formation with regard to the energy level diagrams shown in Figure 7. When FMN accepts an electron and undergoes subsequent protonation to form FMNH^·^, the aromatic system is perturbed, in particular, the central ring. The individual molecular orbitals (MO) are polarized due to the additional electron lifting the degeneracy in energy levels for α and β spins, resulting in a singly occupied molecular orbital (SOMO) for the α electron, which is significantly lower in energy than the original LUMO of FMN. The first two transitions with oscillator strengths greater than 0.05 (D_1_ and D_3_) involve excitations of an electron with β spin and closely resemble the transitions of the ground-state FMN (S_1_ and S_4_) in terms of the involved MOs. However, the reduced fundamental gap results in a red-shifted absorption peak at 591 nm (2.10 eV), as also obtained experimentally.

As can be seen in Figure 7, the SOMO of α is stabilized due to the polarization of the electronic system by the additional electron. This also leads to structural relaxation that not only further stabilizes the SOMO for both spins, but destabilizes the HOMO for β spin due to Coulomb interaction. More specifically, the bond alternation of the benzene ring is reduced from 0.018 Å in FMN to 0.005 Å in FMNH^·^, suggesting an increase in aromaticity. Thus, the small changes in the geometry of FMNH^·^ relative to FMN and the polarization of the electronic system by the additional electron contribute toward the lowering of the excitation energy for the first transition of the β electron, elucidating the red shift of the absorption band. This is a direct consequence of the lifting of the degeneracy of the MO energy levels for the individual electron spins and its impact on the geometry of the flavin system.

The shape of the MOs (see Figure S2) does not drastically change when going from FMN to FMNH^·^ despite the observed changes in the energy of the frontier MOs. The electron spin density (Figure S4) is not evenly distributed over the whole isoalloxazine ring and is most prominent in the middle ring. This is reflected in the chemical shifts, CSAs, and the HFCs in FMN and FMNH^·^. Considering the chemical shifts and CSA tensors of FMN, N5 experiences a large deshielding effect, while exhibiting sp^2^ hybridization. Interestingly, due to its strongly photo-CIDNP-enhanced signal intensity, it suggests high electron spin density in the radical pair state. Similarly, N10 exhibits high nuclear polarization, even though it is not part of the aromatic system. This high electron spin density is further reflected in the HFCs determined for FMNH^·^. In this case, both nitrogens, N5 and N10, exhibit a strong influence on the hyperfine structure while the contributions from N1 and N3 are negligible. Additionally, the large HFC of H5 is an indication of high electron spin density at this particular position. The large anisotropy of the HFCs from both N5 and H5 indicates a localization of the radical electron in the p_*z*_-orbital. The high electron spin density is expected since the light-induced reaction in the wild-type of *Cr*LOV1 involves a bond formation to the sulfur of a highly conserved cysteine at position C4a. Therefore, high electron density around C4a is pivotal for this reaction step, which is reflected in the electron density distribution of the cofactor. Likewise, HFCs of H6 and H8α further suggest an increased electron spin density in the benzene ring of the isoalloxazine, albeit lower compared to the middle ring. This could explain its nucleophilic reactivity observed in select flavoproteins.^71−74^

## Conclusions

We present spectroscopic investigations on two oxidation states of the chromophore of *C. reinhardtii* LOV1 C57S, namely, FMN and FMNH^·^. Due to the embedding in the glassy trehalose matrix, the FMNH^·^ radical is virtually stable at room temperature. To explore the electronic structure of FMN, our approach utilizes the solid-state photo-CIDNP effect, enabling the production of nuclear hyperpolarization in u-^15^N-enriched protein samples. This technique allows for the measurement of chemical shifts and CSA tensors, explaining the stabilization effect of trehalose by the inclusion of the protein into the matrix.

As a byproduct of the photocycle, FMNH^·^ is formed. This radical is characterized by absorption spectroscopy and EPR techniques. In combination with quantum chemical calculations, we show that the red shift of the first absorption band upon one-electron reduction and protonation can be traced back to a reduced fundamental gap, i.e., stabilization of the LUMO and destabilization of the HOMO. This is caused by both electronic polarization and structural relaxation of the flavin system lifting the degeneracy of the MOs between both spins.

Our findings pave the way for highly sensitive spectroscopic experiments of photoactive proteins at ambient temperature in a powder sample. Moreover, the stabilization of the chromophore, its byproducts, or photoinduced intermediates allows for their characterization, which can be combined with quantum chemical analysis. For flavins, in particular, trehalose-embedding opens the door for high-throughput screening of light-activated processes of blue-light receptors, such as cryptochromes, an important candidate for magnetosensitivity in migratory birds.

## Supporting Information Available

Photographs of Petri dish with phototropin trehalose glass before and after illumination; excitation energies excited states; MOs of involved transitions; principal values; ^1^H Mims ENDOR; and hyperfine couplings from ORCA (PDF)
