# Supplementary material for: Red Shift in the Absorption Spectrum of Phototropin LOV1 upon the Formation of a Semiquinone Radical: Reconstructing the Orbital Architecture
Source: J Phys Chem B. 2024 Apr 30;128(18):4344–53. doi: 10.1021/acs.jpcb.4c00397 (PMC11089501; doi:10.1021/acs.jpcb.4c00397)
Supplement: Supplementary file 1 — jp4c00397_si_001.pdf [file jp4c00397_si_001.pdf]

Supporting Information for Publication

Red-shift in the Absorption Spectrum of Phototropin LOV1 upon Formation of Semiquinone Radical: Reconstructing the Orbital Architecture

Patrick Kurle-Tucholski[1], Lisa Köhler[1], Ruonan Qin[1], Ziyue Zhao[1], Christian Wiebeler[1,2], Mantas Šimėnas[3], Andreas Pöppl[4], Jörg Matysik[1,]*

[1] Institut für Analytische Chemie, Universität Leipzig, Linnéstraße 3, D-04103 Leipzig, Germany

[2] Institut für Physik, Universität Augsburg, Universitätsstraße 1, D-86159 Augsburg, Germany

[3] Faculty of Physics, Vilnius University, Sauletekio 3, LT-10257 Vilnius, Lithuania

[4] Felix Bloch Institute for Solid State Physics, Universität Leipzig, Linnéstraße 5, D-04103, Leipzig, Germany

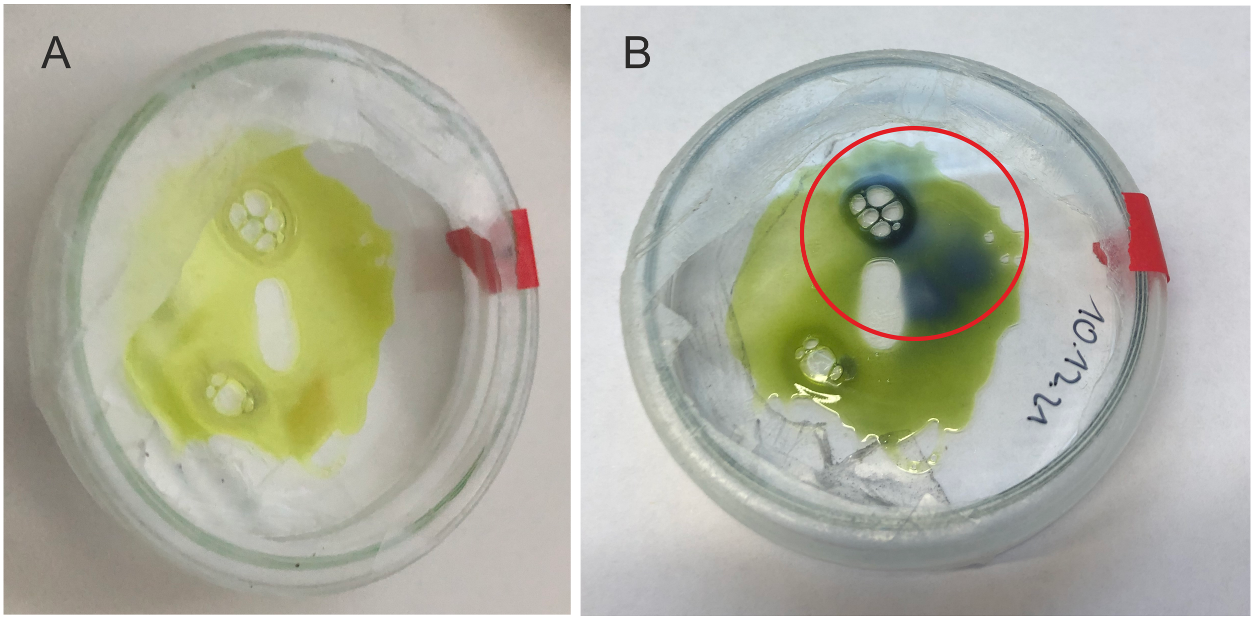

Figure S1: Petri dish containing CrLOV1 incorporated in a glassy sugar matrix. (**A**) Yellow matrix shows FMN in its ground state before illumination. (**B**) The glassy matrix is irradiation with 488-nm CW laser directly inside the red circle. The UV/Vis spectrum was recorded at that point. The color of the surrounding protein-trehalose matrix changed to green indicating a mixture of FMN and FMNH$^{•}$.

Table S1: Transitions, excitation energies in nm and eV, oscillator strengths as well as the MO pair with the highest contribution to the transition together with its weight for the first ten excited states of FMN. The transitions highlighted in bold are shown as sticks in the absorption spectrum of Fig. 2. The first nine transitions are dominated by one pair of canonical MOs, whereas two MO pairs with nearly the same weight contribute to S_{10}. The electron is preferentially excited into the LUMO except for the two highest transitions, where LUMO+1 and LUMO+2 are involved.

Transition	Exc. Energy		Osc. Str.	MO Pair	Weight
	[nm]	[eV]			
**S_1**	**448.5**	**2.765**	**0.200**	**HOMO -> LUMO**	**0.94**
S_2	382.6	3.240	0.000	HOMO-2 -> LUMO	0.79
S_3	375.8	3.300	0.000	HOMO-3 -> LUMO	0.79
**S_4**	**364.2**	**3.405**	**0.272**	**HOMO-1 -> LUMO**	**0.91**
S_5	316.1	3.922	0.000	HOMO-5 -> LUMO	0.98
S_6	308.3	4.021	0.013	HOMO-4 -> LUMO	0.92
S_7	290.0	4.275	0.048	HOMO-6 -> LUMO	0.90
S_8	273.4	4.534	0.000	HOMO-7 -> LUMO	0.99
S_9	266.4	4.654	0.791	HOMO -> LUMO+1	0.88
S_{10}	250.4	4.951	0.087	HOMO -> LUMO+2<br>HOMO-1 -> LUMO+1	0.52<br>0.45

Table S2: Transitions, excitation energies in nm and eV, oscillator strengths as well as the MO pair with the highest contribution to the transition together with its weight for the first ten excited states of FMNH•. The transitions highlighted in bold are shown as sticks in the absorption spectrum of Fig. 2. With the exception of D_9, all transitions are dominated by one pair of canonical MOs and most of the time, an excitation of an electron with β spin is involved.

Transition	Exc. Energy		Osc. Str.	MO Pair	Weight
	[nm]	[eV]			
**D_1**	**591.7**	**2.096**	**0.106**	**HOMO -> SOMO (β)**	**0.96**
D_2	468.0	2.649	0.014	SOMO -> LUMO (α)	0.81
**D_3**	**456.1**	**2.718**	**0.072**	**HOMO-1 -> SOMO (β)**	**0.74**
D_4	421.8	2.939	0.000	HOMO-2 -> SOMO (β)	0.97
**D_5**	**370.9**	**3.343**	**0.083**	**SOMO -> LUMO+2 (α)**	**0.80**
D_6	362.4	3.422	0.027	HOMO-3 -> SOMO (β)	0.85
D_7	351.6	3.526	0.000	HOMO-4 -> SOMO (β)	0.74
D_8	347.4	3.568	0.000	HOMO-5 -> SOMO (β)	0.71
D_9	344.5	3.599	0.015	SOMO -> LUMO+1 (β)<br>HOMO -> LUMO (α)	0.37<br>0.19
**D_{10}**	**338.7**	**3.661**	**0.111**	**HOMO-6 -> LUMO (β)**	**0.71**

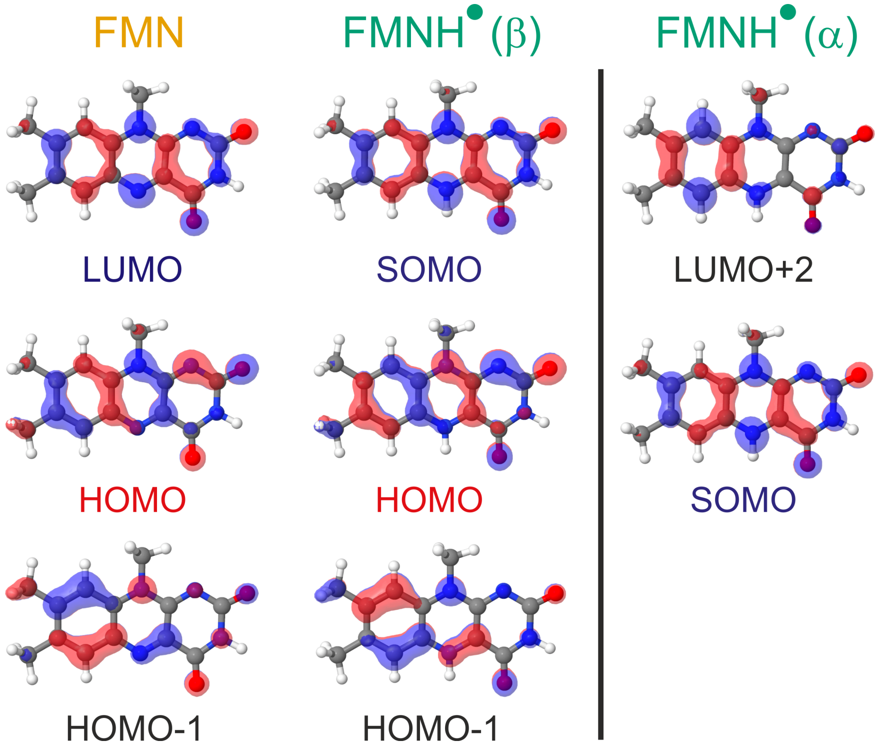

Figure S2: Representation of molecular orbitals (MOs) that are involved in the bright transitions and depicted in the MO scheme.

Table S3: ^{15}N principal values (δ_{11}, δ_{22}, δ_{33}), span (Ω) and asymmetry parameter (η) of the frozen solution (Sol.) and the trehalose glass (TG) for all three occurring signals taken from fitting of CSA tensors shown in Figure 4. Fits were carried out with ssNake 1.3[1].

δ_{iso} [ppm]	Position	δ_{11} [ppm]		δ_{22} [ppm]		δ_{33} [ppm]		Ω [ppm]		η	
		Sol.	TG	Sol.	TG	Sol.	TG	Sol.	TG	Sol.	TG
342	FMN N5	537	539	324	352	166	135	370	404	0.81	0.91
156	FMN N10	218	216	152	151	98	100	120	117	0.86	0.84
129	W N1	156	173	132	131	99	83	57	90	0.79	0.89

Table S4: ^{15}N principal values (δ_{11}, δ_{22}, δ_{33}), span (Ω) and asymmetry parameters (η) for FMN and tryptophan (Trp) obtained from DFT calculations with ORCA. For these calculations, the TPSSh functional and pcSseg-2 basis set with an implicit water solvent model were employed. The absolute shielding constants from the calculations can be converted into chemical shifts by correlating the calculated values with the experimentally determined chemical shifts (see Table S3). From the linear fit (R^2=0.99) the shielding can be converted using the formula $\delta_{calc} = -1.0034 \cdot \delta_{exp} + 238.8$.

Molecule	Position	δ_{11} [ppm]	δ_{22} [ppm]	δ_{33} [ppm]	δ_{iso} [ppm]	Ω [ppm]	η
FMN	N1	322	230	28	193	293	0.56
	N3	185	178	117	160	68	0,16
	N5	671	392	-38	342	709	0.73
	N10	238	188	48	158	190	0.45
Trp	N1	190	116	63	123	127	0.79

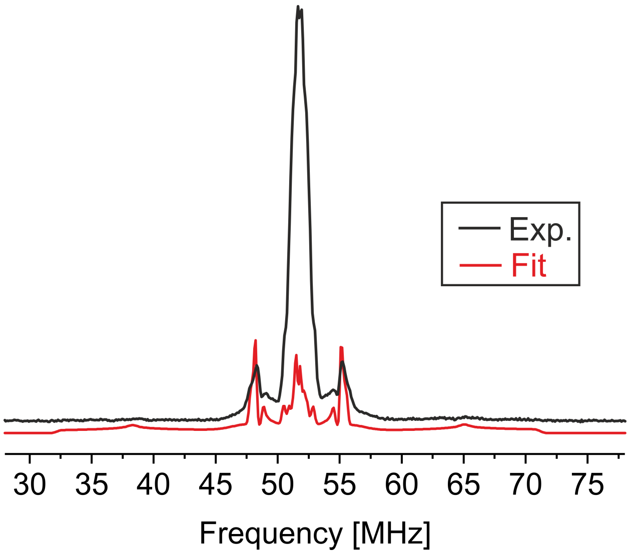

Figure S3: Experimental (black) and simulated (red) Mims ^{1}H ENDOR spectrum at Q-band frequency. For simulation, the ^{1}H HFCs from Table 2 have been used.

Table S5: Hyperfine couplings for $FMNH^•$ in MHz obtained from DFT calculations with ORCA.

Position	A_x	A_y	A_z	A_{iso}
H3	0.5976	-3.4542	-3.5473	-2.1346
H5	-1.9041	-26.7787	-40.8634	-23.1820
H6	-1.6163	-6.3939	-6.6719	-4.8940
H9	-0.3270	0.9162	3.8283	1.4725
H7α	0.1842	-0.4802	0.6658	0.1232
H8α	9.0285	9.2570	11.3904	9.8920
H1’[a]	12.3551	12.6674	16.8414	13.9546
N1	-0.1381	-0.2795	-0.4745	-0.2974
N3	-0.7962	-1.1740	1.6415	-0.1096
N5	-1.7113	-1.8383	47.1892	14.5465
N10	0.1251	-0.1751	26.3454	8.7651

[a] In the DFT calculations, a methyl-group replaced the side-chain of $FMNH^•$. Average of three protons is given here.

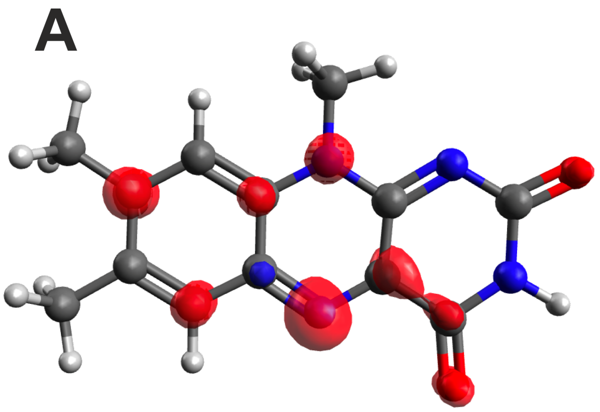

Figure S4: Electron density of (**A**) $FMN^{•-}$ and (**B**) $FMNH^•$ as calculated by ORCA.

Table S6: Fitting parameters for the three identified individual components of W98-N1 in trehalose (Figure 3, top).

δ_{iso} [ppm]	δ_{11} [ppm]	δ_{22} [ppm]	δ_{33} [ppm]	Ω [ppm]
130	171	129	90	81
128	173	131	83	90
125	168	129	76	92

Benchmark of Vertical Excitations

Employing the structures optimized with TPSSh/aug-cc-pVTZ and polarizable continuum model (PCM), we performed further excited state calculations with TD-DFT to assess the influence of the functional and PCM. For this, we employed the Gaussian 16 software suite [2] and in addition to the TPSSh functional with 10 % exact exchange, we employed the PBE0 functional with 25 % exact exchange and the long-range corrected LC-ωPBE functional. From these calculations, we only report the two lowest bright transitions with significant oscillator strength (> 0.020) for both FMN and FMNH$^•$. To summarize the results, the first bright transition of FMN and FMNH$^•$ is always dominated by the excitation from HOMO to LUMO and in case of FMNH$^•$, this applies for the β spin. Similarly, when PCM is employed, the strongest contribution to the second bright transition corresponds to the excitation from HOMO-1 to LUMO, although its weight of 25 % is relatively small for the calculation with the long-range corrected functional.

Table S7: Transitions, excitation energies in nm and eV, oscillator strengths as well as the MO pair with the highest contribution and its weight for the two lowest bright transitions of FMN calculated with three different functionals with and without PCM.

Transition	Exc. Energy		Osc. Str.	MO Pair	Weight*
	[nm]	[eV]			
**TPSSh with PCM**					
S_1	446.1	2.780	0.200	HOMO -> LUMO	0.95
S_4	360.4	3.441	0.248	HOMO-1 -> LUMO	0.91
**TPSSh without PCM**					
S_2	438.0	2.831	0.179	HOMO -> LUMO	0.95
S_6	336.0	3.690	0.100	HOMO-5 -> LUMO	0.87
**PBE0 with PCM**					
S_1	419.1	2.958	0.262	HOMO -> LUMO	0.96

S_3	342.8	3.617	0.266	HOMO-1 -> LUMO	0.93
**PBE0 without PCM**					
S_1	410.7	3.019	0.227	HOMO -> LUMO	0.96
S_5	319.6	3.880	0.139	HOMO-2 -> LUMO	0.88
**LC-ωPBE with PCM**					
S_1	368.7	3.362	0.503	HOMO -> LUMO	0.95
S_3	291.4	4.254	0.168	HOMO-1 -> LUMO	0.90
**LC-ωPBE without PCM**					
S_1	358.0	3.463	0.389	HOMO -> LUMO	0.95
S_4	273.7	4.531	0.089	HOMO-1 -> LUMO	0.81

*The weight for these restricted calculations was determined as twice the squared coefficient.

Table S8: Transitions, excitation energies in nm and eV, oscillator strengths as well as the MO pair with the highest contribution and its weight for the two lowest bright transitions of FMNH$^{\bullet}$ calculated with three different functionals with and without PCM.

Transition	Exc. Energy		Osc. Str.	MO Pair	Weight*
	[nm]	[eV]			
**TPSSh with PCM**					
D_1	598.2	2.073	0.105	HOMO -> SOMO (β)	0.96
D_3	454.0	2.731	0.062	HOMO-1 -> SOMO (β)	0.73
**TPSSh without PCM**					
D_1	627.9	1.975	0.087	HOMO -> SOMO (β)	0.97
D_5	426.1	2.910	0.025	HOMO-2 -> SOMO (β)	0.60
**PBE0 with PCM**					
D_1	564.0	2.198	0.138	HOMO -> SOMO (β)	0.96
D_2	439.2	2.823	0.069	HOMO-1 -> SOMO (β)	0.85
**PBE0 without PCM**					

D_1	590.8	2.099	0.110	SOMO -> LUMO (β)	0.96
D_5	385.0	3.221	0.022	HOMO-3 -> SOMO (β)	0.84
**LC-ωPBE with PCM**					
D_1	500.7	2.476	0.249	SOMO -> LUMO (β)	0.91
D_4	316.2	3.921	0.058	HOMO-1 -> SOMO (β)	0.25
D_5	310.6	3.991	0.111	SOMO -> LUMO+8 (α)	0.38
**LC-ωPBE without PCM**					
D_1	510.5	2.429	0.182	HOMO -> SOMO (β)	0.91
D_5	325.6	3.808	0.031	SOMO -> LUMO+7 (α)	0.25
D_6	310.3	3.996	0.057	HOMO-3 -> SOMO (β)	0.32

*The weight for these unrestricted calculations was determined as the square of the coefficient.

Vibronic Calculations

To model the fine structure of the two lowest energy absorption bands, we performed vibronic calculations for the first two bright states of FMN and FMNH•. For this, we employed the Gaussian 16 software suite [2] together with the default adiabatic Hessian model, i.e. employing optimized geometries and normal modes for the involved electronic states. [3] Based on our previous experiences with such calculations [4] and the results from the benchmark study given above, we decided to employ PBE0/aug-cc-pVTZ with PCM as level of theory for all calculations reported in this section. For the simulation of vibronic spectra, we only included the Franck-Condon terms together with an integral threshold of 10^{11} and employing 8 classes. Absorption spectra were then obtained by convoluting each vibronic transition with Gaussian functions using a half-width at half-maximum of 400 cm^{-1}.

For FMN, we obtained spectrum progressions of around 99 % towards the analytic limit for both S_1 and S_3. Unfortunately, it was not possible to simulate vibronic absorption spectra for FMNH• with this approach, as the spectrum progressions remained rather low for both states D_1 and D_2. Therefore and based on the similarity of the first two bright transitions between FMN and FMNH•, we assumed that the absorption bands of FMNH• may exhibit a similar vibronic progression as FMN. Owing to this and based on vertical excitation calculations with the same level of theory, s. Table S7, we shifted the corresponding absorption bands of FMN by the average shift in excitation energies of the two relevant transitions (6317 cm^{-1}) and scaled their intensities by the ratio of oscillator strengths, i.e. 0.561 and 0.253 for D_1 and D_2, respectively to estimate the absorption bands of FMNH•. The resulting spectra are shown in Figure S5.

In case of FMN, the position of the lowest energy absorption band from the vibronic simulations is slightly blue-shifted relative to experiments, but slightly red-shifted for the second absorption band. Regarding the relative strengths of the vibronic progressions, it is underestimated for the first absorption band, but fits relatively well for the second one. Regarding the absorption bands of FMNH$^{\bullet}$, the mismatch in positions and absolute intensities is larger, which is most likely caused by the employed assumption for approximating these bands. Nonetheless, the relative intensities and positions within a band appear to be rather similar between simulations and experiments. Therefore, the results from these simulations suggest that each of the absorption bands originates from one electronic transition. These transitions are further discussed in the main text based on results from vertical excitation calculations with TPSSh/aug-cc-pVTZ and PCM as level of theory.

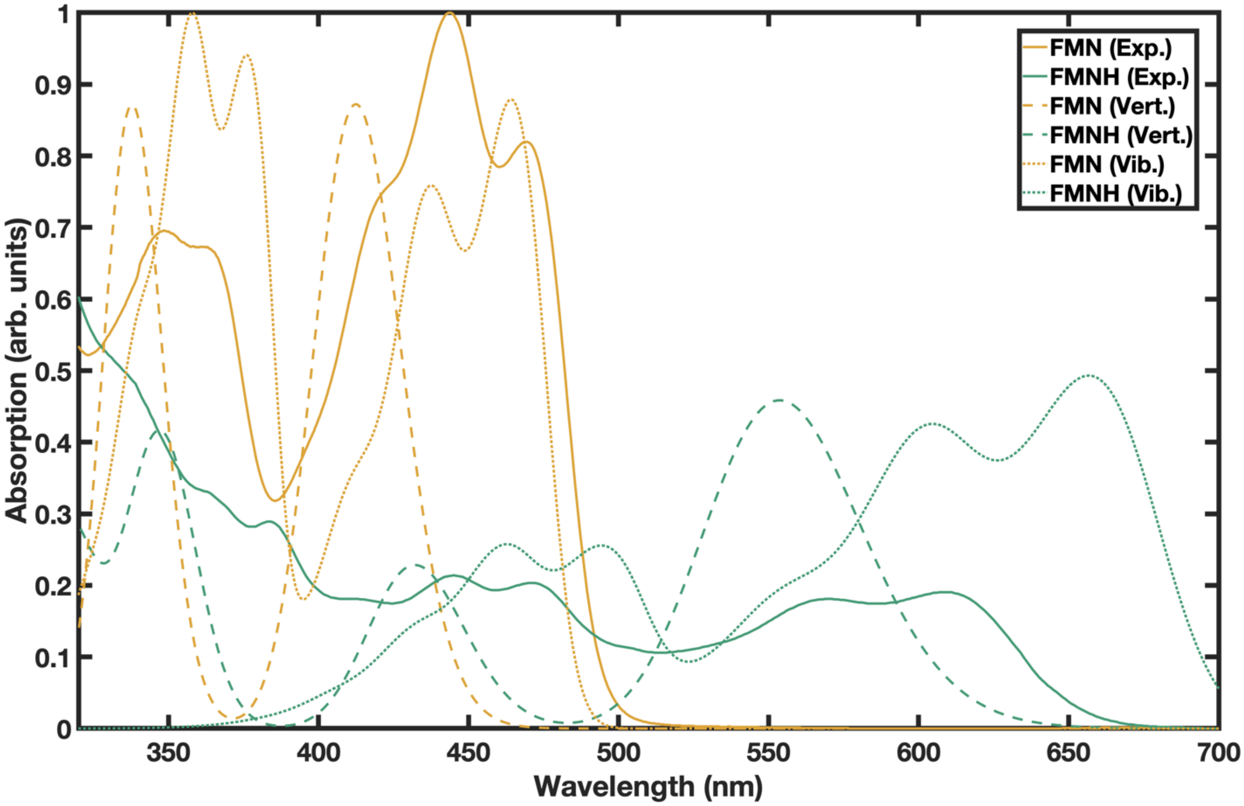

Figure S5: Absorption spectra of FMN (orange) and FMNH$^{\bullet}$ (green) from experiments (solid lines), vertical excitation calculations (dashed lines) and vibronic simulations of the two bright transitions (dotted lines). Intensities were normalized on 1 based on the highest experimental and computed absorption value in the shown spectral range. Please note, that in case of FMNH$^{\bullet}$, the vibronic simulations did not lead to converged spectra, therefore we estimated the corresponding band shapes based on the results of FMN, s. the text and table S7 for further details.

Table S9: Transitions, excitation energies in eV and oscillator strength from vertical excitation calculations of FMN and FMNH$^{\bullet}$. Furthermore, columns 6 and 7 list the shift in excitation energies in eV and cm^{-1}, respectively, and column 8 contains the ratio of oscillator strengths. These latter quantities were used to approximate the absorption band shapes of FMNH$^{\bullet}$ as described in the text.

	FMN		FMNH				
**State**	**Exc. Energy**	**Osc. Str.**	**Exc. Energy**	**Osc. Str.**	**Shift (eV)**	**Shift (cm-1)**	**Ratio of Osc. Str.**
S_1/D_1	3.006	0.2661	2.239	0.1399	0.767	6185	0.526
S_3/D_2	3.670	0.2658	2.871	0.0672	0.800	6449	0.253

```
31
Structure of FMN in xyz format
N     2.55424    -1.29788     0.00008
C     3.73660    -0.61914     0.00008
O     4.83028    -1.18024    -0.00016
N     3.71275     0.78646     0.00008
C     2.60344     1.59038     0.00028
O     2.67458     2.81235    -0.00005
C     1.33081     0.82541     0.00012
N     0.20983     1.49308     0.00008
C    -0.95359     0.79380     0.00005
C    -2.17527     1.49635     0.00001
C    -3.38658     0.84283    -0.00006
C    -4.67647     1.61467    -0.00012
C    -3.39190    -0.58311    -0.00009
C    -4.69373    -1.32986    -0.00018
C    -2.19929    -1.29106    -0.00004
C    -0.96816    -0.62474     0.00004
N     0.23650    -1.29849     0.00008
C     1.42508    -0.61698     0.00011
C     0.23770    -2.77005     0.00006
H     4.61605     1.24610    -0.00006
H    -2.12620     2.57880     0.00002
H    -4.48227     2.68689     0.00005
H    -5.28091     1.37195     0.87844
H    -5.28065     1.37219    -0.87894
H    -5.29149    -1.06674    -0.87777
H    -5.29160    -1.06677     0.87733
H    -4.52949    -2.40672    -0.00019
H    -2.23514    -2.37126    -0.00008
H     1.26926    -3.09907     0.00023
H    -0.27608    -3.12739    -0.89174
H    -0.27637    -3.12739     0.89170
```

32
Structure of FMNH• in xyz format

N	-2.57962	-1.31319	-0.00002
C	-3.76560	-0.64500	0.00002
O	-4.86110	-1.21550	-0.00016
N	-3.74701	0.75760	-0.00005
C	-2.62181	1.54903	-0.00004
O	-2.66376	2.78646	-0.00006
C	-1.40095	0.78989	0.00001
N	-0.21652	1.45846	0.00004
C	0.98586	0.79860	0.00005
C	2.19922	1.49169	0.00005
C	3.41194	0.82096	0.00004
C	4.70256	1.59291	0.00008
C	3.40898	-0.59306	0.00001
C	4.70123	-1.36205	0.00000
C	2.19788	-1.28138	0.00001
C	0.97175	-0.61327	0.00003
N	-0.24753	-1.30041	0.00003
C	-1.44787	-0.61966	0.00000
C	-0.23572	-2.76496	0.00006
H	-4.64624	1.22275	-0.00011
H	2.17339	2.57579	0.00006
H	4.51234	2.66611	-0.00019
H	5.30747	1.34943	-0.87820
H	5.30715	1.34983	0.87870
H	5.30578	-1.11614	0.87814
H	5.30576	-1.11615	-0.87815
H	4.51578	-2.43603	0.00001
H	2.21848	-2.36214	-0.00002
H	-1.26489	-3.10332	0.00017
H	0.27949	-3.12780	0.89056
H	0.27930	-3.12784	-0.89054
H	-0.25449	2.47265	0.00004

References

(1) van Meerten, S. G. J.; Franssen, W. M. J.; Kentgens, A. P. M. ssNake: A cross-platform open-source NMR data processing and fitting application. *J. Magn. Reson.* **2019**, *301*, 56–66. DOI: 10.1016/j.jmr.2019.02.006.

(2) Gaussian 16, Revision C.02, Frisch, M. J.; Trucks, G. W.; Schlegel, H. B.; Scuseria, G. E.; Robb, M. A.; Cheeseman, J. R.; Scalmani, G.; Barone, V.; Petersson, G. A.; Nakatsuji, H.; Li, X.; Caricato, M.; Marenich, A. V.; Bloino, J.; Janesko, B. G.; Gomperts, R.; Mennucci, B.; Hratchian, H. P.; Ortiz, J. V.; Izmaylov, A. F.; Sonnenberg, J. L.; Williams-Young, D.; Ding, F.; Lipparini, F.; Egidi, F.; Goings, J.; Peng, B.; Petrone, A.; Henderson, T.; Ranasinghe, D.; Zakrzewski, V. G.; Gao, J.; Rega, N.; Zheng, G.; Liang, W.; Hada, M.; Ehara, M.; Toyota, K.; Fukuda, R.; Hasegawa, J.; Ishida, M.; Nakajima, T.; Honda, Y.; Kitao, O.; Nakai, H.; Vreven, T.; Throssell, K.; Montgomery, J. A., Jr.; Peralta, J. E.; Ogliaro, F.; Bearpark, M. J.; Heyd, J. J.; Brothers, E. N.; Kudin, K. N.; Staroverov, V. N.; Keith, T. A.; Kobayashi, R.; Normand, J.; Raghavachari, K.; Rendell, A. P.; Burant, J. C.; Iyengar, S. S.; Tomasi, J.; Cossi, M.; Millam, J. M.; Klene, M.; Adamo, C.; Cammi, R.; Ochterski, J. W.; Martin, R. L.; Morokuma, K.; Farkas, O.; Foresman, J. B.; Fox, D. J. Gaussian, Inc., Wallingford CT, 2016.

(3) Barone, V.; Bloino, J.; Biczysko, M.; Santoro, F.; Fully Integrated Approach to Compute Vibrationally Resolved Optical Spectra: From Small Molecules to Macrosystems. *J. Chem. Theory Comput.,* **2009**, *5*, 3, 540-554. DOI: 10.1021/ct8004744

(4) Vollbrecht, J.; Wiebeler, C.; Bock, H.; Schumacher, S.; Kitzerow, H.-S.; Curved Polar Dibenzocoronene Esters and Imides versus Their Planar Centrosymmetric Homologs: Photophysical and Optoelectronic Analysis. *J. Phys. Chem. C*, **2019**, *123*, 7, 4483-4492. DOI: 10.1021/acs.jpcc.8b10730
